# Factors determining hydrocephalus after decompressive craniectomy: the role of interhemispheric hygroma

**DOI:** 10.3389/fneur.2026.1815427

**Published:** 2026-06-22

**Authors:** Andreea-Emanuela Baciu, Ana-M. Castaño León, Sofía Martínez Molina, Alfonso Lagares

**Affiliations:** 1 Department of Neurosurgery, Hospital Universitario 12 de Octubre, Madrid, Spain; 2 Instituto de Investigación Sanitaria Hospital 12 de Octubre (imas12), Madrid, Spain; 3 Departamento de Cirugía, Facultad de Medicina, Universidad Complutense de Madrid, Madrid, Spain

**Keywords:** cerebrospinal fluid dynamics, decompressive craniectomy, post-traumatic hydrocephalus, subdural hygroma, traumatic brain injury

## Abstract

**Introduction:**

Post-traumatic hydrocephalus (PTH) is a recognized complication following decompressive craniectomy (DC) in patients with traumatic brain injury (TBI). Reported risk factors remain inconsistent across studies. Interhemispheric subdural hygroma has been suggested as a potential radiological marker, although its quantitative relevance has not been clearly established. This study aimed to evaluate the association between interhemispheric hygroma and hydrocephalus after DC and to explore whether hygroma thickness may help identify patients at higher risk of treatment requirement.

**Methods:**

We conducted a retrospective observational study at a Level I trauma center including patients older than 15 years who underwent DC for TBI between 2011 and 2025. Radiological hydrocephalus was defined using a modified frontal horn index >33% combined with Gudeman CT criteria. Clinical, surgical, and radiological variables were analyzed. Multivariable logistic regression was used to identify independent associations. The discriminative performance of interhemispheric hygroma thickness for predicting hydrocephalus requiring treatment was assessed using receiver operating characteristic (ROC) curve analysis, and the optimal threshold was determined using the Youden index.

**Results:**

A total of 104 patients were included; 52 (50%) developed radiological hydrocephalus. The median follow-up of the cohort was 13.9 months (IQR 3.8–31.1). Follow-up duration differed significantly between patients with and without radiological hydrocephalus (21 vs. 12.3 months; *p* = 0.013), and between treated and non-treated hydrocephalus (32 vs. 12.4 months; *p* < 0.001). Interhemispheric hygroma thickness was greater in patients with hydrocephalus and remained independently associated in multivariable analysis (OR 1.19 per mm; 95% CI 1.03–1.39). In the subgroup with treated hydrocephalus, hygroma thickness also remained independently associated with treatment requirement (OR 1.30 per mm; 95% CI 1.01–1.67). ROC analysis showed good discriminative performance (AUC 0.81).

**Discussion:**

A threshold of approximately 8 mm demonstrated high sensitivity with acceptable specificity for predicting treatment. Interhemispheric hygroma thickness was independently associated with post-traumatic hydrocephalus after DC and with subsequent treatment requirement. These findings suggest that hygroma thickness may represent a useful radiological parameter for postoperative monitoring, although external validation is warranted.

## Introduction

Nowadays, decompressive craniectomy (DC) is widely accepted in the management of patients with severe traumatic brain injury (TBI) as a surgical procedure intended to treat brain swelling after evacuation of a hemorrhagic mass lesion and/or refractory intracranial hypertension (a third-tier measure according to the Seattle Consensus) (1). Nevertheless, its benefits as a life-saving intervention may be overshadowed by the risk of developing complications related to the DC, including trephinated syndrome, surgical site infections, poor wound healing and cerebrospinal fluid (CSF) disorders, such as hygromas and post traumatic hydrocephalus (PTH) (2–4).

Substantial efforts have been made over the last decades to elucidate the underlying mechanisms and natural history of DC-related hydrocephalus. Although several risk factors have been described in the literature, their association with this complication remains inconsistent (3). One factor deserving particular attention is subdural effusion or hygroma. Initially, it was considered a predictable collateral effect of this surgery rather than a true complication (3). However, although it most often resolves spontaneously, it may act as an early radiological and/or clinical marker of altered cerebrospinal fluid (CSF) dynamics (5–7).

On the one hand, some patients experience neurological deterioration associated with a large hygroma exerting mass effect, occasionally requiring surgical evacuation. On the other hand, some studies have suggested that hygroma may precede the onset of hydrocephalus. Specifically, Kaen et al. (8) published a series of 73 patients with TBI in which interhemispheric hygroma was the only factor independently associated with the development of hydrocephalus by means of multivariable analysis, rather than other commonly studied variables such as subarachnoid hemorrhage, intraventricular hemorrhage, or brain herniation through the cranial defect. This was the first report drawing attention to this specific hygroma location, and the authors also proposed a pathophysiological hypothesis to explain this phenomenon and the genesis of hydrocephalus.

According to this hypothesis, the cerebral falx is initially subjected to significant pressure from brain parenchyma toward the contralateral side. Following decompression, the bony defect accommodates the displaced parenchyma, leaving a potential dead space that becomes filled with CSF, which cannot be adequately resorbed due to mechanical and/or inflammatory obstruction. As CSF circulation remains altered because of the persistent cranial defect—initially necessary to alleviate intracranial pressure—CSF may progressively accumulate within the ventricular system, leading to ventricular enlargement and/or clinical consequences. However, the authors did not assess whether a specific width threshold could be used to identify patients at higher risk of developing hydrocephalus.

We hypothesized that there may be a hygroma size threshold beyond which the risk of developing hydrocephalus increases and which should prompt closer clinical and radiological follow-up. Therefore, this study had two main objectives:

1. To confirm whether subdural hygroma, with special emphasis on interhemispheric location, is an independent risk factor for hydrocephalus in a larger and independent cohort of patients with TBI treated with DC.
2. To investigate which thickness threshold could better discriminate patients at a higher risk of developing hydrocephalus.

## Methods

We conducted a retrospective observational study at our I-level trauma center. The Department of Neurosurgery records every TBI patient requiring hospitalization in a local registry.

For the purposes of this study, we included into the analysis TBI patients older than 15 years old who underwent DC, either as a primary or secondary procedure, between January 2011 and November 2025.

**Figure 1:**
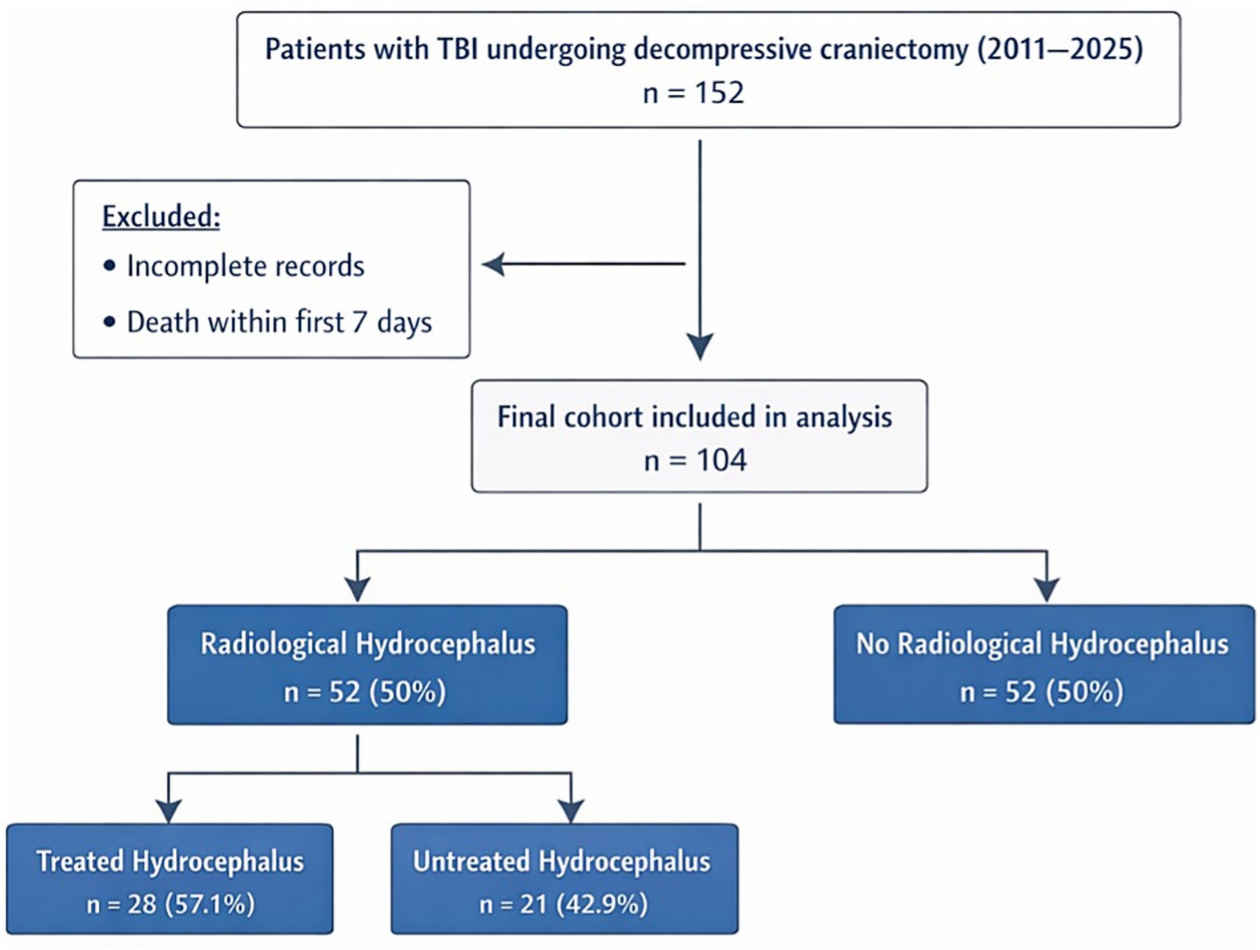
Patient selection flow diagram.

Patients with incomplete medical, surgical, or radiological records were excluded, as well as those who died within the first 7 days after injury, since the likelihood of developing posttraumatic hydrocephalus within such a short period was considered to be very low. A detailed flowchart of the study population, exclusion process, patients included in final analysis and group comparisons is shown in Figure 1.

All patients were managed according to current international guidelines for TBI (1). DC was indicated in cases of diffuse unilateral or bilateral cerebral swelling, intraoperative brain swelling after evacuation of an intracranial hemorrhagic lesion, or as a secondary procedure in patients with refractory intracranial hypertension.

For the analyses, we selected variables considered relevant to the study objectives and previously reported in the literature on PTH. Specifically, we collected demographic data (sex, age, mechanism of injury, presence of extracranial injuries), neurological status at first medical contact and after resuscitation, and surgical variables (craniectomy location—frontotemporoparietal, bicoronal, or bilateral—, performance of duroplasty, use of external ventricular drainage, wound infection, craniectomy diameters, and craniectomy area).

Regarding radiological parameters and the development and management of hydrocephalus, the following variables were recorded:

### Radiological data

**Figure 2:**
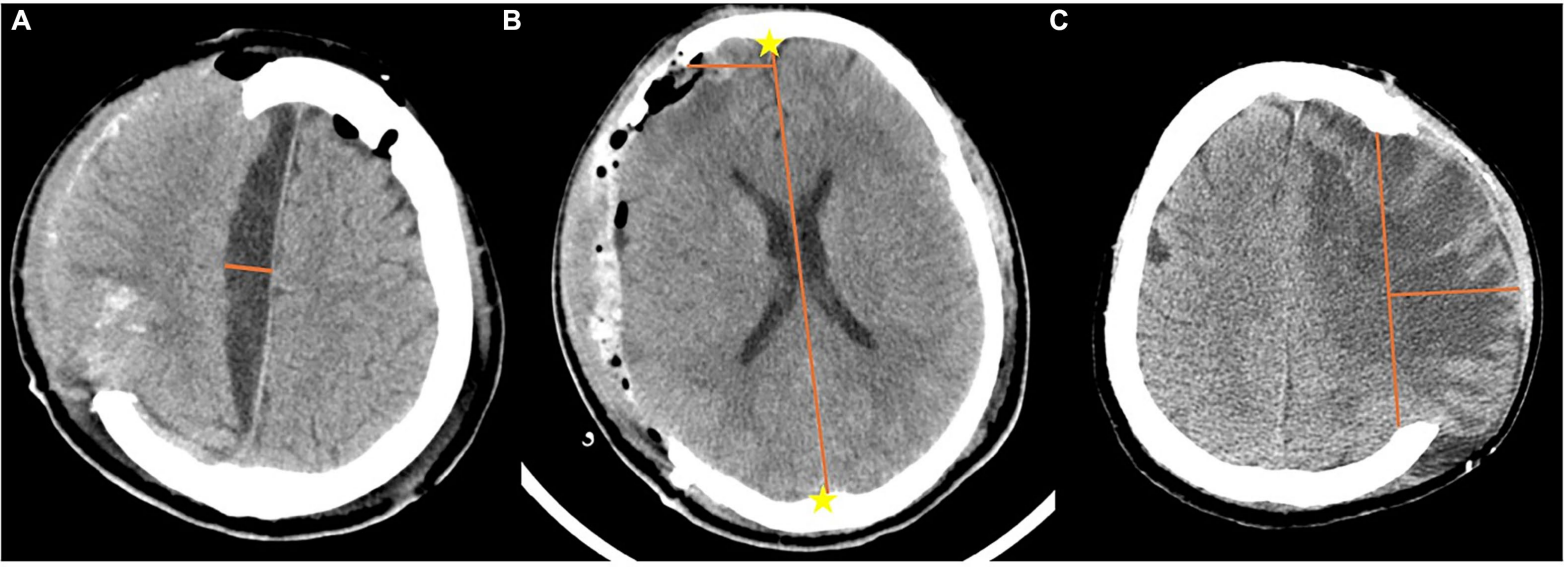
Radiological measures. **(A)** Interhemispheric hygroma; **(B)** distance between craniectomy margin and midline; **(C)** brain herniation.

- Marshall CT classification of the main findings on admission CT scans.
- Presence and extent of traumatic subarachnoid hemorrhage.
- Presence of intraventricular hemorrhage.
- Posttraumatic hydrocephalus, defined according to a previous study as the presence on follow-up CT scans of both of the following criteria (2, 8):

1. A modified Frontal Horn Index (mFHI)—defined as the maximum width of the frontal horns divided by the bicortical diameter at the same axial level—greater than 33%.
2. Gudeman CT criteria: dilatation of the frontal horns of the lateral ventricles with enlargement of the temporal horns and the third ventricle.

- Subdural hygromas (Figure 2A), postsurgical hypodense subdural collections, classified according to their location in relation to the craniectomy (ipsilateral, contralateral, bilateral, or interhemispheric). For this variable, we recorded the CT scan in which the hygroma was first identified, its maximum thickness in millimeters (mm), and whether surgical management was required. Maximum hygroma thickness was measured on axial CT scans by identifying the slice showing the maximum width of the hygroma. Once the slice with the greatest collection was selected, the maximum thickness was measured as the largest perpendicular distance across the hygroma.
- Distance between the craniectomy margin and the anatomical midline (Figure 2B), which was defined as the linear distance (in centimeters-cm-) measured on axial CT from the medial edge of the craniectomy defect to the theoretical sagittal plane of the skull based on fixed cranial landmarks, e.g., falx cerebri, irrespective of any midline shift secondary to mass effect.
- Brain herniation through the craniectomy defect (Figure 2C). This is measured linearly on axial-CT using a method that is relatively standardized in the TBI literature. First, we selected the axial slice showing maximum parenchymal protrusion. In the same slice, we identified the original bony plane by drawing an imaginary line connecting the osseus margins of the craniectomy defect and a perpendicular line was drawn from this plane to the point of maximal outward parenchymal bulging. The distance was measured in centimeters (cm). Classically, herniation is considered significant when the protrusion exceeds 1.5 cm. This cutoff point has been associated with a higher risk of complications, including disturbances in CSF dynamics and hydrocephalus (4, 7, 9).

All follow-up CT scans performed during the postoperative period were reviewed for the analysis of radiological variables. We considered the follow up period as the time between surgery and last CT available for every patient.

### Management of hydrocephalus

Hydrocephalus-specific treatment was indicated for patient with confirmed enlargement of ventricles through radiological indexes and showing radiological progression and/or clinical worsening not explained by other causes. Management included temporary measures (external ventricular drainage or external ventricular shunt) or definitive treatment (ventriculoperitoneal shunt, cranioplasty, or both).

### Statistical analysis

Categorical variables are presented as absolute and relative frequencies. Continuous variables are reported as medians with interquartile ranges, except for those showing a normal distribution according to the Kolmogorov–Smirnov or Shapiro–Wilk tests, which are presented as means with standard deviations.

Two main analytical scenarios were defined for group comparisons:

- Presence versus absence of radiological hydrocephalus.
- Treated versus untreated hydrocephalus.

First, a univariable analysis was performed to identify factors associated with the development of each hydrocephalus scenario, with particular emphasis on the influence of interhemispheric hygroma. Group comparisons were conducted using the chi-square test for categorical variables and the Mann–Whitney *U* test or Student’s *t*-test for continuous variables, as appropriate.

Variables showing a *p-*value < 0.05 in the univariable analysis were subsequently included in a multivariable analysis using binary logistic regression. All selected variables were entered simultaneously into the model. In multivariable analysis, variables showing a *p*-value < 0.05 were considered to be significant.

The discriminative ability of interhemispheric hygroma thickness for predicting hydrocephalus requiring treatment was assessed using the area under the receiver operating characteristic (ROC) curve (AUC), and the optimal threshold was defined as the point maximizing the Youden Index (sensitivity + specificity – 1).

Finally, we performed a secondary analysis to assess the impact of hydrocephalus on patient outcomes. Specifically, we evaluated its effect on in-hospital mortality and 1 year outcome, as measured by the Glasgow Outcome Scale Extended (GOSE). The GOSE scale was dichotomized into unfavorable (1–4) and favorable (5–8) outcomes to facilitate clinically meaningful interpretation and to ensure statistical robustness given the sample size. Analyses were adjusted for baseline severity using the IMPACT-predicted mortality for in-hospital mortality and the IMPACT-predicted functional prognosis for functional outcome.

All statistical analyses were performed using SPSS Statistics version 25.0 for macOS (IBM Corp., Armonk, NY, USA). Statistical significance was defined as a two-sided *p-*value < 0.05.

## Results

### Study population

A total of 152 patients with TBI who underwent DC between January 2011 and November 2025 were initially identified. After applying the exclusion criteria (incomplete records and/or exitus within the first 7 days), the final cohort comprised 104 patients eligible for analysis (Figure 1).

The median age in the whole cohort was 35 years old (IQR 25–52). Globally, there was a majority of male patients (76.9%, 80 patients). 59.6% (62 patients) underwent primary DC, whereas 42 patients (40.4%) had this treatment in the setting of refractory intracranial hypertension. Median length of hospital stay after DC was 40 days (IQR 28–59 days). Median follow up of the whole cohort was 13.9 months (IQR 3.8–31.1 months). Follow-up duration differed significantly between groups. Patients who developed radiological hydrocephalus had longer follow-up compared to those who did not (21 months [IQR 6.18–47.33] vs. 12.3 months [IQR 1.45–21.2]; *p* = 0.013). Similarly, patients requiring treatment for hydrocephalus had longer follow-up than non-treated patients (32 months [IQR 8.8–56] vs. 12.4 months [IQR 1.8–21.48]; *p* < 0.001).

Baseline demographic, clinical, radiological and surgical characteristics of the study population are summarized in Tables 1, 2 according to the two main analytical scenarios which were considered, as aforementioned.

**Table 1:** Radiological hydrocephalus.

	No. of patients (%)			*p*
	Total	With hydrocephalus	Without hydrocephalus	
No. of patients Variable	104	52 (50)	52 (50)	
Age *Median (IQR)*	35 (25–52)	37 (28–53)	30 (37–54)	0.917
Sex *n (%)*				0.352
Masculine	62 (59.6)	42 (80.8)	38 (73.1)	
Feminine	42 (40.4)	10 (19.2)	14 (26.9)	
Marshall CT classification *n (%)*				0.529
Type II	16 (15.4)	6 (11.5)	10 (19.2)	
Type III	11 (10.6)	6 (11.5)	5 (9.6)	
Type IV	7 (6.7)	5 (9.6)	2 (3.9)	
Type V	64 (61.5)	33 (63.5)	31 (59.6)	
Type VI	6 (5.8)	2 (3.9)	4 (7.7)	
Subarachnoid hemorrhage *n (%)*				0.5
Presence	95 (91.3)	47 (90.4)	48 (92.3)	
Absence	9 (8.7)	5 (9.6)	4 (7.7)	
Thickness SAH *n (%)*				0.823
Fine	27 (25)	13 (25)	14 (26.9)	
Thick	77 (75)	39 (75)	38 (73.1)	
Intraventricular hemorrhage *n (%)*				0.202
Presence	32 (30.8)	19 (36.5)	13 (25)	
Absence	72 (69.2)	33 (63.5)	39 (75)	
Type of decompression *n (%)*				0.230
Primary	62 (59.6)	34 (63.4)	38 (73.1)	
Secondary	42 (40.4)	18 (34.6)	14 (26.9)	
Craniectomy *n (%)*				0.171
Frontotemporal	90 (86.5)	47 (90.3)	43 (82.7)	
Bifrontal	13 (12.5)	4 (7.8)	9 (17.3)	
Bilateral	1 (1)	1 (1.9)	0 (0)	
Decompression area (cm^2^) *Mean (SD)*	111.6 (22.7)	112.9 (21.2)	114.4 (22)	0.710
Herniation > 1.5 cm	16 (15.3)	5 (31.3)	11 (68.8)	0.103
Distance midline-craniectomy (cm) *Median (IQR)*	3.85 (3.2–4.7)	3.75 (3.2–4.7)	4 (3.3–4.7)	0.394
Duraplasty *n (%)*	77 (93.9)	39 (50.6)	38 (49.4)	0.522
Hygroma *n (%)*	79 (76)	41 (51.9)	38 (48.1)	0.491
Time to hygroma development *Median (IQR)*	3 (1–8)	3 (1.5–8)	3 (1–9)	0.648
Hygroma location *n (%)*				0.099
Ipsilateral	19 (24.1)	9 (21.9)	10 (26.3)	
Contralateral	3 (3.8)	3 (7.3)	0 (0)	
Bilateral	10 (12.7)	3 (7.3)	7 (18.4)	
Interhemispheric	47 (59.5)	26 (63.5)	21 (55.3)	
Maximum IHH hygroma thickness (mm) *Median (IQR)*	8.32 (5.4–12.9)	11.32 (7.7–14.8)	6.26 (4.5–10.0)	0.009

**Table 2:** Treated hydrocephalus.

	No. of patients (%)			
	Total	Treated hydrocephalus	Untreated hydrocephalus	*p*
No. of patients Variable	49	28 (57.1)	21 (42.9)	
Age *Median (IQR)*	37 (28–53)	43 (28–58)	40 (26–53)	0.551
Sex *n (%)*				0.565
Masculine	39 (79.6)	22 (78.6)	17 (81)	
Feminine	10 (20.4)	6 (21.4)	4 (19)	
Marshall CT classification *n (%)*				0.109
Type II	5 (10.2)	5 (17.9)	0 (0)	
Type III	5 (10.2)	3 (10.7)	2 (9.5)	
Type IV	5 (10.2)	3 (10.7)	2 (9.5)	
Type V	32 (65.3)	15 (53.6)	17 (81)	
Type VI	2 (4.1)	2 (7.1)	0 (0)	
Subarachnoid hemorrhage *n (%)*				0.099
Presence	44 (89.8)	27 (96.4)	17 (81)	
Absence	5 (10.2)	1 (3.6)	4 (19)	
Fine SAH *n (%)*	13 (26.5)	7 (25)	6 (28.6)	0.779
Thick SAH *n (%)*	36 (73.5)	21 (75)	15 (71.4)	
Intraventricular hemorrhage *n (%)*				0.669
Presence	18 (36.7)	11 (39.3)	7 (33.3)	
Absence	31 (63.3)	17 (60.7)	14 (66.7)	
Type of decompression *n (%)*				0.93
Primary	33 (67.3)	19 (67.9)	14 (66.7)	
Secondary	16 (32.7)	9 (32.1)	7 (33.3)	
Craniectomy *n (%)*				0.549
Frontotemporal	46 (93.9)	26 (92.8)	20 (95.2)	
Bifrontal	2 (4.1)	1 (3.6)	1 (4.8)	
Bilateral	1 (2)	1 (3.6)	0 (0)	
Decompression area (cm^2^) *Mean (SD)*	112 (20.9)	109 (22.4)	114.7 (19.5)	0.359
Herniation > 1.5 cm *n (%)*	5 (10.2)	3 (60)	2 (40)	
Distance midline-craniectomy (cm) *Median (IQR)*	3.75 (3.2–4.7)	4.1 (3.3–4.8)	3.4 (3.2–4.4)	0.289
Duraplasty *n (%)*	38 (95)	22 (57.9)	16 (42.1)	0.508
Hygroma *n (%)*	39 (79.6)	22 (56.4)	17 (43.6)	0.680
Time to hygroma development	3 (1.5–8)	3 (1.75–8)	3 (0.75–8.25)	0.861
Hygroma location *n (%)*				0.310
Ipsilateral	9 (23.1)	4 (18.2)	5 (29.4)	
Contralateral	3 (7.7)	1 (4.5)	2 (11.8)	
Bilateral	2 (5.1)	2 (9.1)	0 (0)	
Interhemispheric	25 (64.1)	15 (68.2)	10 (58.8)	
Maximum IHH hygroma thickness (mm) *Median (IQR)*	11.3 (7.7–14.8)	12.9 (8.4–16.7)	7.7 (5.7–11.5)	0.01
Time from decompression (days)	43 (23–116)	50 (31–153)	29 (18–98)	0.031

### Radiological hydrocephalus

A total of 52 patients (50%) fulfilled criteria for radiological hydrocephalus (Figure 1). The diagnosis of PTH was established in the first follow-up CT in which both criteria (mFHI and Gudeman) were present. There were no significant differences in terms of demographic characteristics between both subgroups. In regard of initial findings on CT, according to Marshall CT classification, there were no significant differences between both groups. Subarachnoid hemorrhage (SAH) was an almost ubiquitous finding in all initial CT’s (95 patients, 91.3%) but, although non-significant, there was a trend towards thick SAH being more common in patients who did not develop radiological hydrocephalus (90.4% vs. 92.3%). However, intraventricular hemorrhage was more frequent in patients that had radiological hydrocephalus (36.5%). This trend was also not significant.

Secondary DC was relatively more frequent in patients who did not present with radiological hydrocephalus (73.1% vs. 63.4%, *p* = 0.230). Patients without radiological hydrocephalus had had more bifrontal craniectomies than those with hydrocephalus (9 patients vs. 4 patients). Bilateral decompression was performed in only one patient in the cohort, who was later diagnosed with radiological hydrocephalus. There was no significant difference in the location of decompression.

Contrary to expectations, traditional factors associated with the development of post-traumatic hydrocephalus showed a different pattern in our patient cohort. Patients with radiological hydrocephalus had smaller bone defect areas, a lower rate of brain herniation >1.5 cm, and a greater distance from the midline to the medial edge of the craniectomy. Nevertheless, none of these factors reached statistical significance in the comparison between groups.

**Figure 3:**
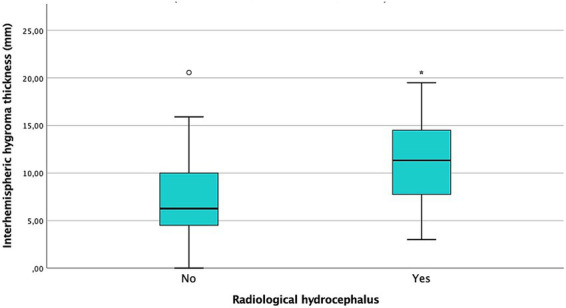
Boxplot distribution of interhemispheric hygroma thickness in patients with and without radiological hydrocephalus. *Interhemispheric hygroma size was significantly larger in patients developing hydrocephalus (11.32 mm vs 6.26 mm,* *p* *= 0.009)*.

In most of our cohort, the presence of hygroma could be detected in postsurgical radiological controls (79 patients, 76%). The median time from DC to hygroma detection did not differ significantly between patients without radiological hydrocephalus (3 days [IQR 1–9]) and those who developed radiological hydrocephalus (3 days [IQR 1.5–8]; *p* = 0.48) (Table 1). Moreover, while ipsilateral hygroma was more frequent in patients that did not develop hydrocephalus, (26.3% vs. 21.9%), interhemispheric hygroma was significantly more common in hydrocephalus group (63.5% vs. 55.3%). These trends were not significant (*p* = 0.099). Notably, interhemispheric hygroma size was significantly larger in patients developing hydrocephalus (11.32 mm vs. 6.26 mm, *p* = 0.009) (Figure 3).

For this scenario, the multivariable analysis included all variables with a *p* < 0.05 and also other classical PTH-associated radiological variables (DC area, herniation > 1.5 cm, distance to midline, hygroma location). However, the variable that remained significant was the maximum interhemispheric hygroma width (*p* = 0.022). Interhemispheric hygroma thickness had an OR of 1.19 (IC95% 1.026–1.39) (Table 3), indicating a 19% increase in the odds of developing radiological hydrocephalus per millimeter increase.

**Table 3:** Multivariable logistic regression analysis for factors associated with radiological hydrocephalus.

Variable	Odds ratio (OR)	95% confidence interval	*p*-value
Maximum interhemispheric hygroma thickness (per mm)	1.19	1.03–1.39	0.022
Hygroma location	NA	NA	<0.99
DC area (per cm^2^)	0.98	0.95–1.02	0.303
Distance DC-midline	1.19	0.7–2.00	0.53
Herniation > 1.5 cm	NA	NA	<0.99

Binary logistic regression model including variables with *p* < 0.05 in univariable analysis and classical PTH-associated radiological variables. All variables were entered simultaneously into the model. OR, odds ratio; CI, confidence interval; NA, non-available. Maximum interhemispheric hygroma thickness was the only variable reaching statistical significance in the multivariable model (*p* = 0.022).

### Treated hydrocephalus

In this scenario, only the radiological hydrocephalus subset of patients was taken into account. There was complete information for 49 out of 52 patients (94%). There was no difference in the basal characteristics of the cohort and trends in age, sex, initial Marshall CT classification, type and location of decompression were similar as in the previous scenario and can be seen in Table 2. Twenty-eight patients (57.1%) underwent treatment for hydrocephalus (Figure 1). Six patients (21.4%) received temporary treatment (5 with EVD -17.9%- and 1 with externalized ventricular shunt −3.6%-). Twenty-two patients (78.6%) had a definitive treatment for PTH. The most frequent surgery was VP shunt + cranioplasty (11 patients, 39.2%), followed by VP shunt only (9 patients, 32.1%) and cranioplasty only (27.2%). SAH and intraventricular hemorrhage were more common in patients with treated hydrocephalus, but this was not significant.

**Figure 4:**
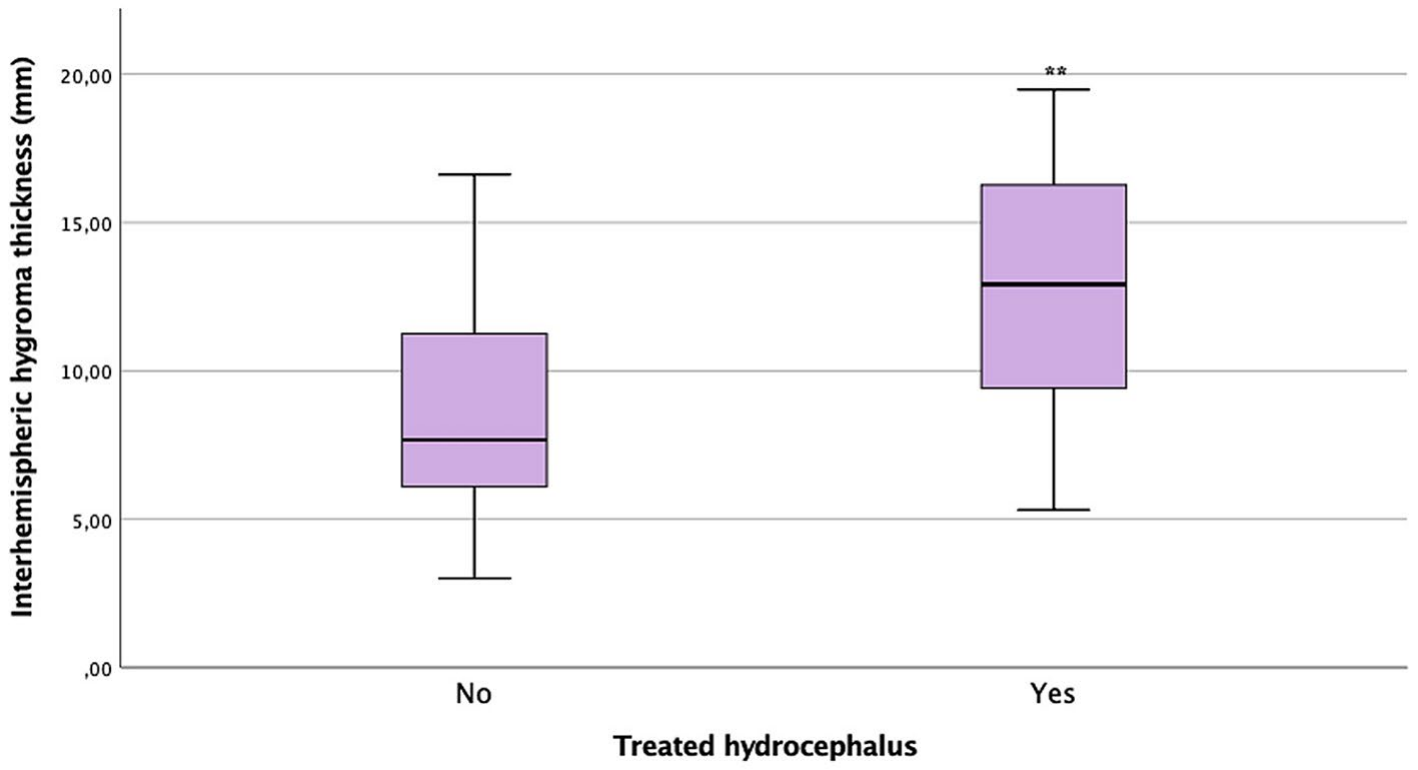
Boxplot distribution of interhemispheric hygroma thickness according to the necessity of hydrocephalus treatment. *The maximum interhemispheric hygroma width continued to be higher in patients that were treated for hydrocephalus in contrast to those who were untreated (12.9 mm vs 7.7 mm, *p* = 0.01)*.

The mean decompression area was 109 cm^2^ (SD 22.4) in treated patients and 114.7 cm^2^ (SD 19.5) in untreated ones (non-significant). On the other hand, patients who were not treated for hydrocephalus had more frequently ipsilateral hygroma (6 vs. 4 patients), but interhemispheric continued to be the most common hygroma location in both groups, predominantly in treated patients (68.2% vs. 58.2%). Although the hygroma location differences were non-significant in this scenario, the maximum interhemispheric hygroma width continued to be higher in patients that were treated for hydrocephalus in contrast to those who were untreated (12.9 mm vs. 7.7 mm, *p* = 0.01) (Figure 4). There was not a statistically significant difference in the time to hygroma diagnosis between patients who were treated for hydrocephalus (3 days – IQR 1.75–8 days) and those who were not (3 days [IQR 0.75–8.25 days]) (*p* = 0.861) (Table 2).

Regarding the timing of hydrocephalus diagnosis after the decompression, patients who were treated for this complication used to have a more delayed development of hydrocephalus compared to patients that were not treated (50 days vs. 29 days), and this difference was significant (*p* = 0.031).

For this scenario, the multivariable analysis included all variables with a *p* < 0.05. Accordingly, the only variable that remained significant was the maximum interhemispheric hygroma width (*p* = 0.039). Interhemispheric hygroma width had an OR of 1.3 (95%CI 1.01–1.668) (Table 4), meaning that each mm of interhemispheric hygroma was associated with a risk of 30% for receiving treatment in patients with radiological hydrocephalus.

**Table 4:** Multivariable logistic regression analysis for factors associated with hydrocephalus requiring treatment.

Variable	Odds ratio (OR)	95% confidence interval	*p-*value
Maximum interhemispheric hygroma thickness (per mm)	**1.30**	**1.01–1.67**	**0.039**
Time from decompression (per day)	1.00	0.99–1.01	0.971

Binary logistic regression model including variables with *p* < 0.05 in univariable analysis. All variables were entered simultaneously into the model. OR, odds ratio; CI, confidence interval. Maximum interhemispheric hygroma thickness was the only variable reaching statistical significance in the multivariable model (*p* = 0.039).

**Figure 5:**
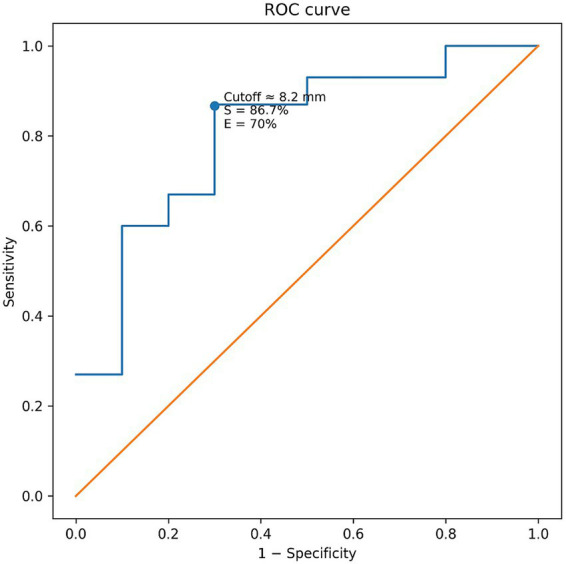
Receiver operating characteristic (ROC) curve for interhemispheric hygroma thickness predicting hydrocephalus requiring treatment. *The area under the curve was 0.81 (95% CI 0.63–0.99), indicating good discriminatory ability. The highlighted point corresponds to a threshold of approximately 8.2 mm, yielding a sensitivity of 86.7% and a specificity of 70%.*

In the aim of finding the threshold that had a more accurate predictive value, by means of receiver operating characteristic (ROC) curve (Figure 5), an interhemispheric hygroma of at least 8.2 mm approximately (8.185 mm), had an 86.7% sensitivity and a 70% specificity for the need of post decompression hydrocephalus treatment. Receiver operating characteristic analysis demonstrated good discriminatory ability of interhemispheric hygroma thickness for predicting hydrocephalus requiring treatment (AUC 0.81, 95% CI 0.63–0.99; *p* = 0.011).

### Outcomes in patients with hydrocephalus

Finally, we performed a secondary analysis to assess the prognostic impact of hydrocephalus across the prespecified subgroups, including in-hospital mortality and 1-year functional outcome as measured by the GOSE scale.

According to the presence or absence of radiological hydrocephalus, in-hospital mortality was 5.8% (3 patients) in those with radiological hydrocephalus and 11.5% (6 patients) in those without it. After adjustment for IMPACT-predicted mortality, radiological hydrocephalus was not independently associated with in-hospital mortality (OR = 0.469, 95% CI 0.11–1.99; *p* = 0.488).

Similarly, in-hospital mortality in patients who required treatment for hydrocephalus was 7.1% (2 patients), compared to 4.5% (1 patient) in those who did not. After adjustment for IMPACT-predicted mortality, the need for hydrocephalus treatment was not independently associated with in-hospital mortality (OR = 0.758, 95%CI 0.148–3.889; *p* = 0.545) (Table 5).

**Table 5:** Association between hydrocephalus and in-hospital mortality.

Subgroups	In-hospital mortality *n* (%)	Adjusted OR*	95% CI	*p*-value
Radiological hydrocephalus				
No	6 (11.5%)	Reference	**—**	**—**
Yes	3 (5.8%)	0.469	0.11–1.99	0.488
Hydrocephalus treatment				
No	1 (4.5%)	Reference	—	—
Yes	2 (7.1%)	0.758	0.15–3.89	0.545

*Adjusted for IMPACT-predicted mortality using binary logistic regression. OR, odds ratio; CI, confidence interval.

Regarding functional outcome at 1 year, we evaluated both prespecified scenarios: the presence or absence of radiological hydrocephalus and the need for surgical treatment. The distribution of GOSE categories at 1 year according to the presence of radiological hydrocephalus is shown in Table 6. Patients with radiological hydrocephalus exhibited a higher proportion of unfavorable functional outcomes, particularly driven by a higher frequency of lower severe disability (58.8% vs. 26.0%) and a lower proportion of moderate disability (3.9% vs. 16.0%) compared to those without hydrocephalus. In the adjusted analysis including IMPACT-predicted functional prognosis, radiological hydrocephalus was associated with higher odds of unfavorable outcome; however, this did not reach statistical significance (OR = 4.674, 95%CI 0.92–23.6; *p* = 0.063).

**Table 6:** Functional outcome at 1 year (GOSE) according to radiological hydrocephalus.

GOSE at 1 year	No radiological hydrocephalus *n* (%)	Radiological hydrocephalus *n* (%)	Adjusted OR (95% CI)	*p*-value
GOSE categories				
Unknown	2 (4%)	2 (3.9%)		
Death	10 (20%)	6 (11.8%)		
Vegetative state	0 (0%)	2 (3.9%)		
Lower severe disability	13 (26%)	30 (58.8%)		
Upper severe disability	17 (34%)	9 (17.6%)		
Moderate disability	8 (16%)	2 (3.9%)		
Dichotomized GOSE				
Unfavorable outcome (GOSE 1–4)	23 (46%)	47 (92.2%)	4.674 (0.92–23.6)	0.063
Favorable outcome (GOSE 5–8)	25 (50%)	2 (3.9%)	Reference	**—**

Adjusted for IMPACT-predicted functional prognosis (binary logistic regression). The OR refers to the odds of unfavorable outcome (GOSE 1–4) in the presence of radiological hydrocephalus.

The distribution of GOSE categories at 1 year according to the need for hydrocephalus treatment is shown in Table 7. Patients requiring treatment for hydrocephalus showed a higher proportion of unfavorable outcomes compared to non-treated patients (82.1% vs. 61.9%) mainly driven by a higher frequency of lower severe disability (67.9% vs. 47.6%). However, after adjustment for IMPACT-predicted functional prognosis, hydrocephalus requiring treatment was not independently associated with functional outcome (OR = 1.749, 95%CI 0.34–9.09; *p* = 0.506).

**Table 7:** Functional outcome at 1 year (GOSE) according to the performance of hydrocephalus treatment.

GOSE at 1 year	Non treated hydrocephalus *n* (%)	Treated hydrocephalus *n* (%)	Adjusted OR (95% CI)	*p*-value
GOSE categories				
Unknown	1 (4.8%)	0 (0%)		
Death	2 (9.5%)	4 (14.3%)		
Vegetative state	1 (4.8%)	0 (0%)		
Lower severe disability	10 (47.6%)	19 (67.9%)		
Upper severe disability	7 (33.3%)	3 (10.7%)		
Moderate disability	0 (0%)	2 (7.1%)		
Dichotomized GOSE				
Unfavorable outcome (GOSE 1–4)	13 (61.9%)	23 (82.1%)	1.749 (0.34–9.09)	*p* = 0.506
Favorable outcome (GOSE 5–8)	7 (33.3%)	2 (7.1%)	Reference	**—**

Adjusted for IMPACT-predicted functional prognosis (binary logistic regression). The OR refers to the odds of unfavorable outcome (GOSE 1–4) in patients requiring treatment for hydrocephalus.

## Discussion

The reported incidence of post-traumatic hydrocephalus after DC varies widely across studies, ranging from less than 1% to nearly 30% (10), largely depending on diagnostic criteria, timing of assessment, and whether radiological ventriculomegaly or clinically relevant hydrocephalus is considered. This variability has long been recognized and was highlighted by Marmarou et al. (2), who emphasized the difficulty of distinguishing cerebral atrophy from true hydrocephalus using imaging alone.

In the present study, we deliberately adopted classical radiological diagnostic criteria, including ventricular enlargement assessed by the frontal horn index in combination with Gudeman CT criteria, consistent with the methodology used by Marmarou and subsequently by Kaen et al. (2, 8). This approach was chosen to ensure comparability with seminal studies and to avoid overestimation of hydrocephalus by excluding cases of isolated ventriculomegaly or brain atrophy. Despite this conservative definition, the incidence of radiological hydrocephalus in our cohort was 50%, underscoring the high vulnerability of patients undergoing DC and reinforcing the need for reliable markers of clinically meaningful disease.

Beyond confirming the association between subdural hygroma and post-traumatic hydrocephalus, our findings refine the clinical relevance of hygroma location and thickness after DC. Earlier studies describing postoperative subdural hygromas largely focused on ipsilateral collections beneath the cranial defect, often interpreting them as frequent but benign sequelae of surgery. In these series, hygromas were commonly reported in terms of presence rather than anatomical distribution, and interhemispheric collections received comparatively little attention (8, 9).

Kaen et al. (8) were the first to specifically highlight the interhemispheric location as a critical factor, reporting that interhemispheric hygroma preceded the development of hydrocephalus in most affected patients and emerged as the only independent predictor in their multivariable analysis. A recent review also underscores the role of hygroma location (3). Our results confirm the relevance of this specific location in an independent and larger cohort and extend previous observations by demonstrating that interhemispheric hygroma thickness carries prognostic value. This quantitative refinement supports the concept that interhemispheric hygromas are not merely a variant of postoperative fluid collection, but rather a radiological expression of altered cerebrospinal fluid dynamics with distinct clinical implications.

Importantly, this distinction may help explain why ipsilateral hygromas—although more frequently reported in earlier series (5, 6)—have shown inconsistent or weak associations with hydrocephalus, whereas interhemispheric collections appear more closely linked to pathological CSF circulation. From a mechanistic perspective, the anatomical constraints of the interhemispheric space and its proximity to the falx may predispose these hygromas to interfere with CSF absorption and redistribution, rendering their size and evolution more clinically relevant than their mere presence.

Interestingly, traditional radiological variables classically associated with post-traumatic hydrocephalus (7) showed a divergent pattern in our cohort. Patients who developed hydrocephalus had smaller craniectomy areas, lower rates of external cerebral herniation >1.5 cm, and greater distances from the midline to the medial defect margin, although none of these differences reached statistical significance. This apparently paradoxical observation suggests that morphometric characteristics of the decompressive procedure may not directly translate into predictable alterations in postoperative CSF dynamics. However, these findings should be interpreted cautiously in light of the sample size and the possibility of residual confounding. It is plausible that structural parameters interact with complex biomechanical and physiological processes—including intracranial compliance, pressure redistribution, and CSF pulsatility (2, 7)—such that their isolated effect may be insufficient to independently predict ventricular enlargement.

This interpretation is reinforced by multivariable analysis. After adjustment for classical radiological variables—including craniectomy area, distance to midline, external herniation, and hygroma location—maximum interhemispheric hygroma thickness remained the sole independent predictor of radiological hydrocephalus. The observed direct relationship, corresponding to a 19% increase in the odds of hydrocephalus per millimeter increment, supports the internal consistency of this association. Interhemispheric hygroma thickness may therefore represent a radiological surrogate of evolving CSF redistribution and impaired absorption dynamics.

One of the most relevant findings of this study is the identification of a quantitative threshold for interhemispheric hygroma thickness associated with the need for hydrocephalus treatment. In multivariable logistic regression analysis, interhemispheric hygroma thickness remained the only independent predictor of hydrocephalus requiring treatment (OR 1.30 per millimeter increase; 95% CI 1.01–1.67), supporting a dose–response relationship between hygroma size and hydrocephalus risk. Receiver operating characteristic analysis demonstrated good discriminative performance (AUC 0.81), and a threshold of approximately 8 mm yielded high sensitivity (86.7%) with acceptable specificity (70%), suggesting potential utility as an early risk stratification marker rather than as a definitive diagnostic cutoff.

From a clinical perspective, prioritizing sensitivity over specificity appears appropriate, as delayed recognition of clinically relevant hydrocephalus may have greater consequences than intensified surveillance. Thus, this threshold should be interpreted as a tool to guide closer follow-up rather than as an indication for immediate intervention. The observed dose–response relationship further supports the biological plausibility of the association.

Although follow-up duration differed between groups, with longer follow-up observed in patients who developed hydrocephalus and in those requiring treatment, the impact of this difference on detection is probably limited. Most cases were identified within the first year after injury, with only three diagnoses beyond this period. The longer follow-up observed in treated patients likely reflects closer surveillance due to implanted devices, rather than a true difference in disease trajectory. In addition, the timing of hygroma detection was similar across patients subgroups and an early finding, further suggesting that differences in overall follow-up duration, although statistically significant, are unlikely to have had a major influence on the observed associations. Importantly, this temporal pattern aligns with our findings on interhemispheric hygroma and supports the notion that post-traumatic hydrocephalus represents delayed but still early complication in the post-injury course, likely driven by early alterations in CSF dynamics. This temporal relationship further reinforces the concept of a continuum between interhemispheric hygroma and hydrocephalus, both reflecting different stages of the same underlying disturbance in CSF circulation.

Another relevant complication after DC is the potential development of the syndrome of the trephined (SoT), a distinct clinical entity which may be driven by different underlying mechanisms. One proposed explanation involves alterations in cerebral blood perfusion related to the cranial defect, which have been associated with regional cortical changes demonstrated by nuclear imaging (11, 12). Other authors, such as Láng et al. (13), have suggested that both SoT and PTH may represent delayed complications arising from disturbances in cerebrospinal fluid dynamics following DC. However, while both conditions might share a common base of CSF dysregulation following decompressive craniectomy, the present study focused only on factors influencing the arise of PTH.

Several limitations should be acknowledged. First, the retrospective design limits causal inference and introduces potential selection and information bias, despite predefined radiological criteria and systematic follow-up. Second, the single-center nature and moderate sample size may have reduced statistical power to detect weaker associations for other established risk factors, such as subarachnoid or intraventricular hemorrhage.

Third, in the treated hydrocephalus analysis, multivariable adjustment was intentionally restricted to variables showing significant associations in univariable testing due to the limited number of outcome events. Including multiple classical radiological variables in a model with 28 treated cases could have resulted in overfitting and unstable estimates. Moreover, traditional factors such as subarachnoid or intraventricular hemorrhage did not demonstrate significant associations in our cohort and may be more closely related to early ventricular enlargement than to progression requiring CSF diversion (3). Accordingly, the model was designed to preserve statistical robustness while evaluating the independent contribution of interhemispheric hygroma thickness. Nevertheless, a potential influence of perioperative ICP management on CSF dynamics and hygroma formation cannot be excluded. As stated in the Methods section, all patients were managed according to established international guidelines for traumatic brain injury, including principles aligned with the Seattle Consensus (1). In addition, perioperative care was delivered within a dedicated neurocritical care unit by a multidisciplinary team experienced in TBI management and intracranial pressure monitoring. Although this is a retrospective cohort, this structured and protocol-driven clinical environment likely reduced variability in perioperative ICP-lowering strategies across patients. Therefore, the potential impact of medical management on CSF dynamics and hygroma formation was likely relatively consistent within our cohort, although a residual effect cannot be entirely ruled out.

In our cohort, neither radiological hydrocephalus nor hydrocephalus requiring treatment emerged as independent predictors of in-hospital mortality after adjustment for baseline severity. This suggests that, in this setting, hydrocephalus may be more closely related to the underlying severity of brain injury than to early mortality itself.

On the other hand, despite the lack of statistical significance, radiological hydrocephalus showed a consistent trend toward worse functional outcomes. In our data, this likely reflects a limitation of purely morphologic diagnostic criteria, as ventricular enlargement alone encompasses a heterogeneous spectrum ranging from ex-vacuo ventriculomegaly to clinically relevant disturbances in CSF dynamics. Moreover, patients in the non-treated group were themselves heterogeneous, including both asymptomatic individuals and patients with poor neurological status who were not considered candidates for intervention. In the absence of hydrodynamic testing, this may have resulted in a mixture of patients with different underlying CSF dynamics, potentially influencing group classification. These findings are in line with the European Consensus Statement on post-traumatic hydrocephalus (14), which highlights the limited strength of the available evidence and the uncertainty surrounding both diagnostic and therapeutic recommendations. In this context, the heterogeneity observed in our cohort reflects real-world clinical practice, where management is often not standardized and treatment decisions are made on an individual basis.

Given the relatively small number of treated patients, we chose to distinguish between radiological hydrocephalus and hydrocephalus requiring treatment, allowing a clearer separation between imaging findings and clinically relevant disease. However, this distinction must be interpreted with caution. Although dynamic CSF studies were not systematically performed in our cohort, our findings support the potential role of hydrodynamic assessments in identifying cases of hydrocephalus with subtle clinical manifestations or symptoms that may otherwise be attributed to traumatic brain injury sequelae, as suggested by Marmarou et al. (2). Nevertheless, while such studies may provide additional diagnostic value, particularly after cranioplasty when intracranial dynamics are more stable (14), their use in the acute phase of TBI remains limited by feasibility and altered intracranial physiology.

Lastly, the analysis of clinical outcomes should be interpreted as secondary to the primary objective of this study. Our main aim was to explore factors associated with the development of hydrocephalus, particularly the role of interhemispheric hygroma as an early radiological marker. In this regard, our findings support the idea that interhemispheric hygroma may reflect early alterations in CSF dynamics and help identify a subgroup of patients who warrant closer follow-up due to an increased risk of developing clinically significant hydrocephalus and potentially requiring treatment.

Finally, although we focused on clinically relevant hydrocephalus requiring treatment, the decision to initiate CSF diversion was based on multidisciplinary clinical judgment rather than standardized hydrodynamic testing, which may have introduced variability. However, this reflects routine practice in severe TBI, where invasive CSF dynamics studies are rarely feasible. Although interhemispheric hygroma thickness remained independently associated with treatment requirement, the limited number of events warrants cautious interpretation of the precise magnitude of effect.

## Conclusion

Our study suggests that interhemispheric subdural hygroma plays a relevant role in the development of post-traumatic hydrocephalus following DC in patients with traumatic brain injury. Our findings indicate that not only the presence, but also the thickness of interhemispheric hygroma is associated with an increased likelihood of developing hydrocephalus, particularly in cases requiring surgical treatment.

## Data Availability

The original contributions presented in the study are included in the article/supplementary material, further inquiries can be directed to the corresponding author.
